# Case Report: Donor-derived Chagas Disease Post–heart Transplant After False-negative Donor *Trypanosoma cruzi* Testing

**DOI:** 10.1097/TXD.0000000000001864

**Published:** 2025-10-03

**Authors:** Marisa Mihori, Alexandra Bryson, Christopher Doern, Freba Farhat, Guanhua Lai, Mark Mochel, Megan K. Morales, Melissa Smallfield, Inna Tchoukina, Nicole C. Vissichelli

**Affiliations:** 1 Virginia Commonwealth University, School of Medicine, Richmond, VA.; 2 Department of Pathology, Virginia Commonwealth University, Richmond, VA.; 3 Department of Dermatology, MedStar Georgetown University Hospital, Washington, DC.; 4 Division of Infectious Diseases, Department of Internal Medicine, Virginia Commonwealth University, Richmond, VA.; 5 Division of Cardiology, Department of Internal Medicine, Virginia Commonwealth University, Richmond, VA.

Chagas disease (CD) is caused by the protozoan *Trypanosoma cruzi* and it affects 8–10 million people worldwide and >300 000 people in the United States.^1^ It is transmitted primarily by triatomine insects and is the third most common parasite transmitted via organ transplantation.^2^ In heart transplant recipients (HTRs), reactivation of chronic or donor-derived CD (ddCD) can cause severe disease if treatment is delayed, with 75% transmission and 50% mortality.^3^ HTRs carry the highest risk for ddCD due to the parasite’s tropism to cardiac tissue.^4^ Since the early 2000s, 9 cases of transplant-associated transmission have been reported in the United States.^3^ Due to the risk of both reactivation and donor-derived infection, it is recommended that all recipients and donors from endemic areas be screened to mitigate transmission risk.^5^

We present an HTR with ddCD diagnosed after a false-negative donor *T cruzi* serologic screening test. To our knowledge, successful treatment with prolonged survival after symptomatic CD contracted from heart transplant (HT) has not been previously reported. This patient provided informed consent for publication of this case. As a case report, this was exempt from approval by an institutional review or ethics board.

## CASE DESCRIPTION

**FIGURE 1.**
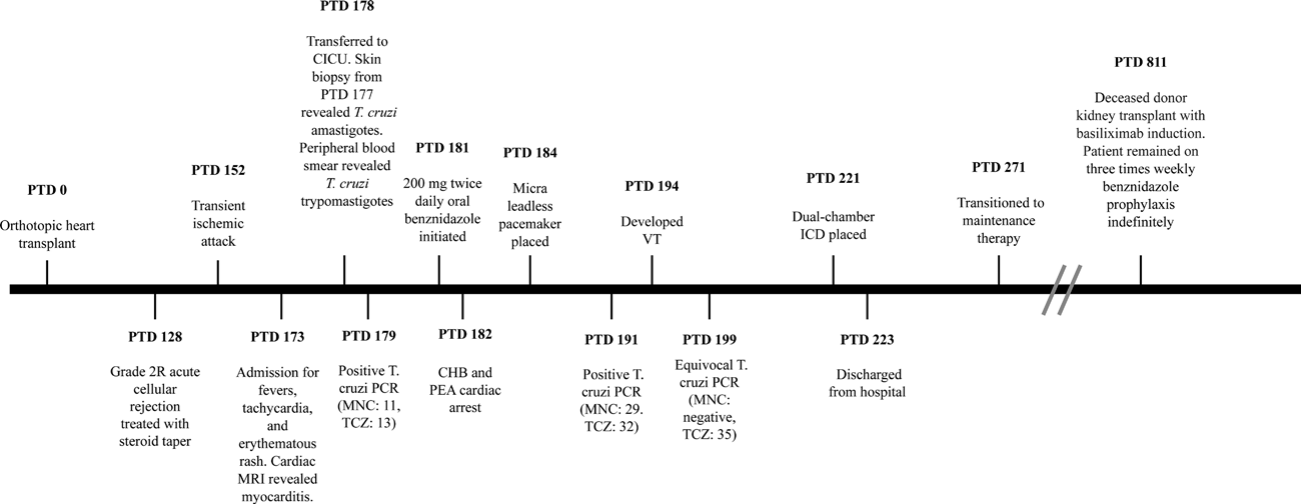
Timeline of significant events along the patient’s clinical course, beginning at cardiac transplant (PTD 0), including *Trypanosoma cruzi* PCR testing results with cycle threshold values. Events are not drawn to scale. CHB, complete heart block; CICU, cardiac intensive care unit; ICD, implantable cardioverter defibrillator; MNC, minicircle kDNA; PCR, polymerase chain reaction; PEA, pulseless electrical activity; PTD, posttransplant day; TCZ, *Trypanosoma cruzi*–specific kinetoplastid; VT, ventricular tachycardia.

A man in his 60s with ischemic cardiomyopathy underwent orthotopic HT with basiliximab induction, atovaquone and valganciclovir prophylaxis, and maintenance immunosuppression with tacrolimus, mycophenolic acid, and prednisone. His posttransplant (PT) course was complicated by grade 2R acute cellular rejection (ACR) treated with steroids and a transient ischemic attack (Figure 1).

**FIGURE 2.**
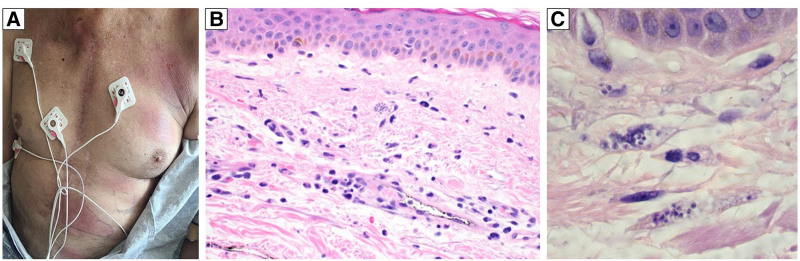
Image of the patient’s well-circumscribed, erythematous rash as it appeared 178 d post–heart transplant (A) and right arm skin biopsy, representative of the disseminated chest rash, collected 177 d post–heart transplant, showing dermal edema, interstitial lymphohistiocytic inflammation, and rare aggregates of amastigotes (B, ×400, H&E), which are present in the cytoplasm of macrophages (C, ×1000 with oil immersion, H&E). H&E, hematoxylin and eosin.

On PT day (PTD) 173, the patient presented for surveillance endomyocardial biopsy (EMBx), was febrile and tachycardic, and was admitted for evaluation. In the preceding week, he had fevers, chills, nonproductive cough, sore throat, and painless, well-demarcated, circular erythematous plaques over his chest (Figure 2). Laboratory tests showed pancytopenia (hemoglobin 10.8 g/dL, 13 000 white blood cells/µL, 77 000 platelets/µL), elevated troponin (3.59 ng/mL), and elevated B-type natriuretic peptide (11 331 pg/mL). EMBx showed grade 1R ACR with reduced interstitial lymphocytic infiltrate and no myocyte damage. Electrocardiogram demonstrated a new right bundle branch block and left anterior fascicular block. Cardiac MRI revealed left ventricular (LV) ejection fraction 42%, mid-anterior akinesis, basal septal hypokinesis, increased LV wall thickness, extensive abnormal myocardial enhancement in the mid-anterior wall and interventricular septum, and exudative pericarditis. A brain MRI demonstrated acute ischemia in the left temporoparietal lobe.

**FIGURE 3.**
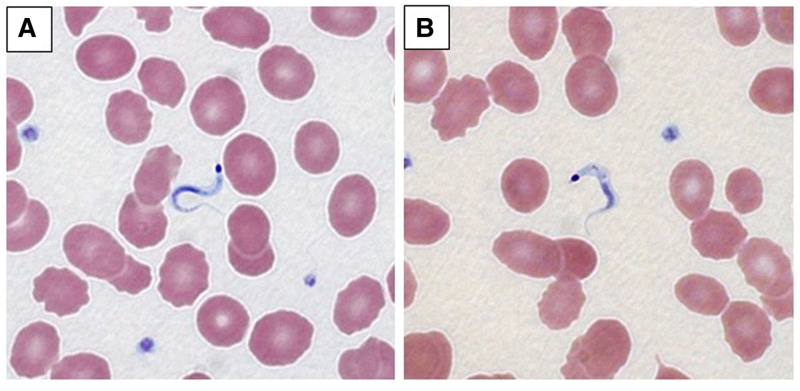
Peripheral smear collected on day 178 post–heart transplant showing *Trypanosoma cruzi* trypomastigotes with characteristic C and S shapes and conspicuous intracellular kinetoplasts (A and B, ×1000 magnification; Wright-Giemsa Stain).

The patient was transferred to the intensive care unit with ongoing fevers and rising troponin, aspartate aminotransferase, and creatinine. Additional infectious workup was negative. A skin biopsy collected on PTD 177 revealed *T cruzi* amastigotes (Figure 2). Peripheral blood smear on PTD 178 revealed prominent *T cruzi* trypomastigotes, which were confirmed by Centers for Disease Control (CDC) Parasitic Diseases Reference Laboratory analysis (Figure 3; **Video 1, SDC,** https://links.lww.com/TXD/A800). *Trypanosoma cruzi* real-time polymerase chain reaction (PCR; CDC) at PTD 179 returned positive.^6^ Oral benznidazole 200 mg twice daily was started. *Trypanosoma cruzi* PCR and peripheral blood smears were monitored weekly, with a negative peripheral smear at 7 d and an equivocal PCR at 21 d. Further testing was delayed because of the suspension of testing at the Parasitic Diseases Reference Laboratory, but all subsequent tests returned negative results.

Donor-derived *T cruzi* infection was suspected as the patient had no travel to endemic areas, whereas the donor was from Guatemala. After the diagnosis was made, *T cruzi* antibody ELISA (Chagatest Recombinant version 0.3.0; Wiener Laboratories, Rosario, Argentina) and immunoblot using trypomastigote-secreted antigens^1^ were tested in saved recipient blood collected 1 d before transplant and the results were negative. At the time of organ procurement, the donor *T cruzi* IgG was negative using the Hemagen enzyme immunoassay (EIA; Hemagen Chagas Kit EIA; Hemagen Diagnostics Inc, Columbia, MD). After infection was detected in the patient, the saved donor serum was tested at CDC with ELISA^2^ and the trypomastigote-secreted antigen immunoblot^1^ had positive results on both tests. In the recipient, amastigotes were not detected on histopathologic review of the explanted native heart nor in any EMBx after transplant.

The patient’s hospital course was complicated by a complete heart block and pulseless electrical activity cardiac arrest requiring pacing and ventricular tachycardia requiring defibrillator implantation. He developed fever, elevated interleukin-2, hyperferritinemia, shock liver, and acute renal failure that progressed to end-stage renal disease. He was discharged on hospital day 50 on benznidazole 200 mg twice daily with mycophenolate held. After completing 90 d of treatment, benznidazole was reduced to 200 mg thrice weekly to prevent recurrence. He underwent a deceased donor kidney transplant with basiliximab induction 811 d after HT with intensified monitoring. An echocardiogram 3.5 y post-HT demonstrates an LV ejection fraction of 35%–40%. He is maintained on tacrolimus (target trough 5–7 ng/mL), mycophenolic acid 360 mg twice daily, and prednisone 5 mg/d and has not developed cardiac or kidney rejection. The patient remains on benznidazole prophylaxis without CD reactivation or adverse effects.

## DISCUSSION

Transplant of hearts from chronically infected donors poses the highest risk for ddCD. Screening at-risk donors can prevent transmission, and recommendations call for withholding hearts from seropositive donors and monitoring recipients of other organs PT.^3,7,8^ Our patient developed severe manifestations several months PT after a false-negative donor screening test.

Targeted screening of donors is recommended for CD (Table 1).^8^ Several immunoassays have US Food and Drug Administration approval and clearance to screen blood and organ donors (Table 2).^7,12^ Performance discrepancies may be due to differences in antigen targets, geographic strain variation, immunogenicity, and host characteristics. Although manufacturer-reported sensitivity and specificity are high, real-world data, particularly in the United States, are limited.^13^ The tests were primarily evaluated in the Southern Cone, where *T cruzi* with discrete typing units TcII, TcV, and TcVI predominate.^14^ However, TcI is predominant in Guatemala, where the donor originated.^14^ The reduced sensitivity of the Hemagen test for TcI may explain the false-negative result.^15,16^

**TABLE 1.** Recommendations for screening living and deceased donors

Indications for screening living and deceased donors for Chagas disease
• Children of women born in endemic regions^*a*^ if their birth mother’s serology is positive or unknown • Donors who have resided in an endemic region^*a*^ for >3 mo • Donors who received a blood transfusion in endemic region^*a*^ • Donors who have a previous diagnosis of Chagas disease

^*a*^Trypanosoma cruzi endemic regions: Argentina, Belize, Bolivia, Brazil, Chile, Colombia, Costa Rica, Ecuador, El Salvador, French Guiana, Guatemala, Guyana, Honduras, Mexico, Nicaragua, Panama, Paraguay, Peru, Suriname, Uruguay, and Venezuela.

**TABLE 2.** US FDA approved and cleared tests to screen for *Trypanosoma cruzi* in organ and blood donors

Test		Manufacturer	US FDA status	Sensitivity	Specificity
Ortho *Trypanosoma cruzi* EIA^7^	ELISA	Ortho Clinical Diagnostics, Raritan, NJ	FDA approved in 2006	100%	99%
Abbott Alinity S chemiluminescent assay^9^	ELISA	Abbott Laboratories, Abbott Park, IL	FDA approved in 2010	100%	100%
Elecsys Chagas^10^	ECLIA	Roche Diagnostics, Indianapolis, IN	FDA approved in 2024	100%	100%
Hemagen Chagas Kit EIA^*a*^ ^11^	EIA	Hemagen Diagnostics Inc, Columbia, MD	FDA cleared	100%	97%
Chagatest Recombinant version 0.3.0 ELISA^*a*^ ^11^	ELISA	Weiner Laboratories, Rosario, Argentina	FDA cleared	99%	100%

^*a*^Used to screen the donor in this case.

^*b*^Sensitivity and specificity are based on the package insert and studies listed; however, further independent studies are needed to confirm test performance across different geographic regions.

EIA, enzyme immunoassay; ECLIA, electrochemiluminescence immunoassay; FDA, Food and Drug Administration.

In acute CD, diagnosis is made by detection of the parasite in blood or tissue through PCR or morphological identification. In our case, CD was only suspected and confirmed by peripheral blood smear and skin biopsy after symptoms emerged. It is possible that the ACR on PTD 18 was an earlier manifestation of CD instead of pure rejection, although EMBx immunohistochemistry did not reveal amastigotes. Additional testing was not completed at that time and no serum was saved to determine whether the serum *T cruzi* PCR would have been positive, but in hindsight, this may have resulted in an earlier diagnosis. EMBx histopathological findings of CD can resemble ACR, and the sensitivity of identifying amastigotes on EMBx may be limited by tissue sampling and parasite burden.^17-19^ Previous reports of ddCD with false-negative donor testing emphasize having a high index of suspicion for CD in immunocompromised patients despite negative serology and consider repeat donor testing and recipient screening if pretest probability is high.^20^ Prompt screening, diagnosis, and treatment of ddCD with benznidazole can preserve graft function and prevent the development of severe CD symptoms.^3^

In a US study (2001–2011), *T cruzi* transmission occurred in 75% of seropositive heart donors.^3^ Three cases of ddCD were diagnosed in HTRs 3–8 wk PT; 2 symptomatic patients died within 6 mo of transplant due to heart failure and acute rejection.^3,21^ Another HTR developed neurologic and cardiac symptoms 9 wk PT and died within 3 mo of diagnosis.^22^ Our patient, diagnosed at 24 wk with fulminant disease, survived with benznidazole treatment and maintenance therapy. In patients from endemic areas who undergo solid organ transplantation, long-term suppressive therapy is not routinely recommended to prevent CD reactivation due to the potential side effects (including myelosuppression, hepatotoxicity, dermatitis, peripheral neuropathy, and gastrointestinal disturbances) and because it is unusual to have multiple episodes of reactivation. However, these are often detected by routine PCR sampling before the development of symptoms.^4^ Long-term suppression has been described only in 1 case of recurrent *T cruzi* after lung transplantation.^23^ Benznidazole was successful in preventing further recurrences, but the patient died 97 wk after transplant due to other causes. Due to the severity of the disease in our patient, long-term suppressive therapy was recommended in consultation with experts at the Parasitology Division of CDC, and similarly, the patient did not have any subsequent recurrence or adverse effects.

This is the first reported case of long-term survival after symptomatic ddCD in an HTR, despite a delayed diagnosis in the setting of a false-negative donor serologic screen. With inconsistent organ donor screening, the potential for infected donors may be underestimated and identifying and screening high-risk patients is crucial.^12^ Using seropositive donors requires timely and stringent peripheral smear and PCR surveillance for early detection and treatment of *T cruzi*. This case highlights the current limitations of serologic testing of CD, as well as the importance of vigilance with transplanting from at-risk seronegative donors and rigorous PT monitoring in at-risk recipients. Further research is needed to optimize management strategies for HTR with CD.

## ACKNOWLEDGMENT

The authors express their sincere gratitude to Susan Montgomery, DVM, MPH, from the Parasitic Diseases Branch of the CDC, for her invaluable expertise and guidance in managing this patient, coordinating diagnostic testing, and providing a critical review of the article.

## Supplementary Material


